# Trapped between darkness and heat: desert birds (*Argya squamiceps*) initiate daily activity earlier (and terminate it later) in response to heat

**DOI:** 10.1098/rspb.2025.0979

**Published:** 2025-11-12

**Authors:** Yitzchak Ben Mocha, Itamar Ring, Oded Keynan

**Affiliations:** ^1^ Center for the Advanced Study of Collective Behaviour, University of Konstanz, Konstanz, Germany; ^2^ Zukunftskolleg, University of Konstanz, Konstanz, Germany; ^3^ Dead Sea and Arava Science Center, Neve Zohar, Israel

**Keywords:** Arabian babbler, cooperative breeding, activity patterns, heat stress, predation risk, conservation, sleep, *Argya squamiceps*, climate change

## Abstract

Diurnal desert endotherms must adjust the timing of their daily activity to mitigate physiological stress from ambient heat. Understanding the limits of this behavioural plasticity is important for predicting how climate change may affect desert species. Here, we studied the onset and termination of daily activity in wild birds (Arabian babblers *Argya squamiceps*) inhabiting the Arava Desert. We found that the birds (i) began/terminated their daily activity within a 45 min window around morning/evening civil twilights, (ii) advanced/delayed the onset/termination of daily activity in response to rising ambient heat, yet (iii) were never active in darkness. These temporal adjustments may help to mitigate energetic deficits caused by heat stress and concurrently extend foraging times under cooler temperatures. Though, subsequent analyses of the climate in the Arava Desert demonstrate (iv) a ~2°C increase in summer temperature over the past 31 years, and (v) that Arabian babblers advanced the onset of daily activity by 8.8 min during the last 11 summers. We thus propose the existence of a ‘heat versus dark trap’: rising temperatures drive diurnal desert endotherms to increase activity during the cooler pre-dawn and post-dusk times, but the leeway of these temporal adjustments is strictly constrained by nocturnal predation risk and reduced foraging efficiency in poor light conditions.

## 1. Introduction

Endotherms thrive (i.e. are capable of reproducing and living) within their thermal neutral zone (i.e. the ambient temperature range where body temperature is maintained with low energetic requirements and water loss) [1,2]. Considerable deviation from this neutral zone may induce physiological stress [3,4], impair cognitive abilities [5,6] and/or require energy investment for thermoregulation [7]. Non-migratory species thus use diverse behaviours to avoid thermal extremes in habitats where temperatures regularly exceed their thermal neutral zone. For example, diversity of birds (e.g. Gambel’s quail *Callipepla gambelii* [8]*,* little bustard *Tetrax tetrax* [9]) and mammals (e.g. African elephant *Loxodonta Africana* [10], Alpine ibex *Capra ibex* [11]) reduce activity during the midday summer heat.

**Figure 1.**
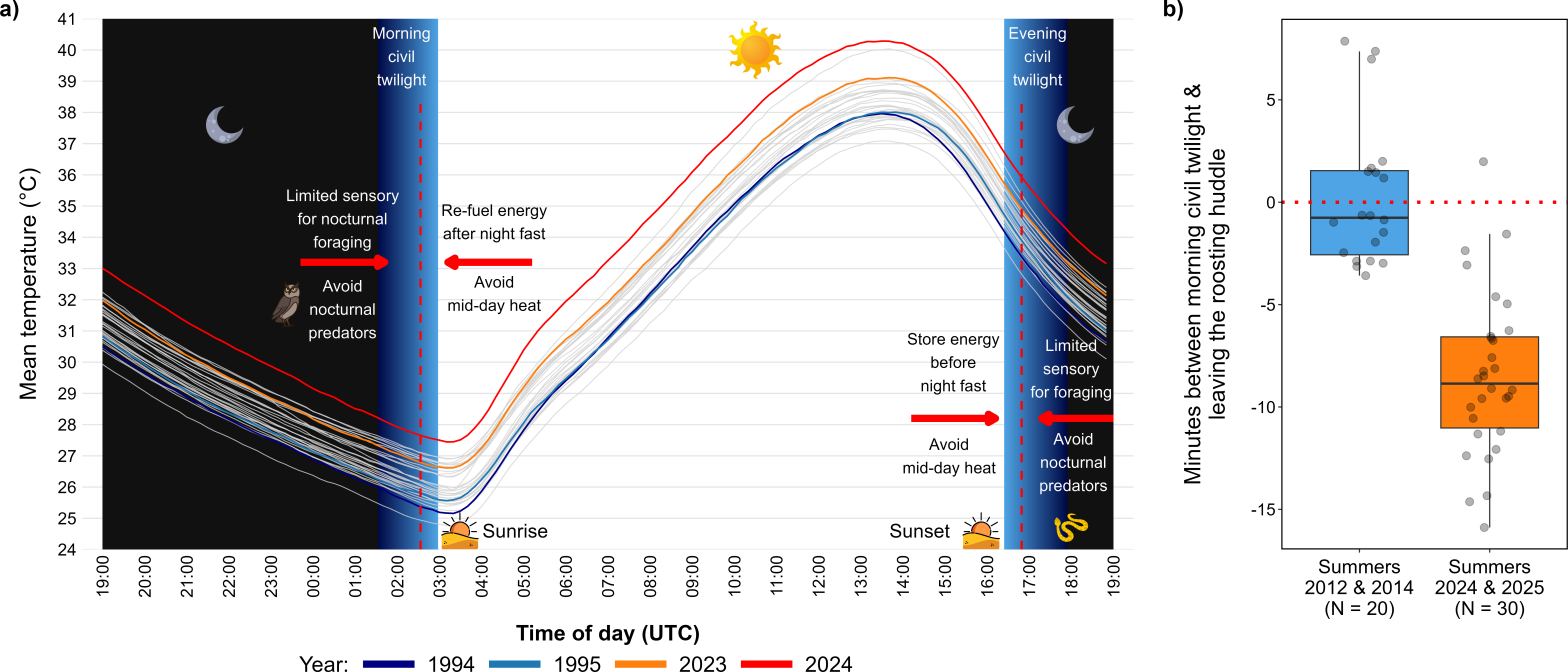
(a) Daily temperature during summer (June–September) by year (1994–2024) at the Shezaf Nature Reserve. Each solid line represents the mean temperature during this hour per summer year. The black-shaded areas represent the night-time and the blue-shaded areas represent the twilight times. The red arrows present suggested selective pressures on the onset and termination times of daily activity. (b) Time leaving the roosting huddle during the first and last summers of the study. The dotted red lines (at *y* = 0) denote the time of morning civil twilights. The horizontal bars represent the median, boxes represent the 25% and 75% quartiles and the vertical lines indicate the minimum and maximum values (points outside the lines are outliers).

Shifting activity to daytime with tolerable temperatures is another key strategy for avoiding and/or mitigating heat stress [12]. This behavioural strategy, though, is only effective if the daytime includes an alternative period long enough to meet the animal’s daily needs within its thermal neutral zone. If alternative periods are too short or if activity shifting is constrained by other factors, for instance, inefficient sensory for nocturnal foraging or higher predation risk [13,14], the animal would have to forage under less favourable temperatures even after maximal temporal adjustment (figure 1) [15,16]. This constraint may be profound for diurnal birds because many of these species naturally initiate daily activity soon after first light [17,18], and night-time may therefore present a natural barrier for further advancing the onset of their daily activity.

The warming of deserts due to climate change presents a further increasing challenge to behavioural strategies for heat mitigation [19,20]. Importantly, temperatures do not need to rise year-round for non-migratory species to be excluded from desert habitats. Instead, further intensification of heat may render the hot season (usually summers) too harsh or too prolonged for native species to survive to the next year [21,22]. For example, several desert birds (e.g. southern yellow-billed hornbills *Tockus leucomelas* [16], southern pied babblers *Turdoides bicolor* [7]) and mammal (e.g. dwarf mongooses *Helogale parvula* [12]) species fail to maintain sustainable body mass during hot summers. Understanding the factors determining the timing of daily activity and the limitations of temporal adjustment of daily activity is crucial for assessing the impact of climate change on species survival [23,24].

Two hypotheses about adjustment of onset and termination of diurnal species’ daily activity in response to ambient temperature are as follows. According to the ‘heat hypothesis’, animals adjust their activity times to mitigate the negative effects of previously experienced heat (e.g. body mass loss [7,25]) and/or to avoid the negative effects of anticipated heat (e.g. inefficient foraging [7,11]). If this hypothesis holds, the animals are predicted to (i) start their daily activity earlier, and (ii) terminate it later, after experiencing heat. These adjustments may help overcome energetic deficiencies as early as possible after the nightly fast, and to fill energetic reserves as late as possible before the night fast begins in the evening. Such temporal adjustments also increase foraging under the cooler dawn and dusk times (figure 1a) [11,12]. In contrast, the ‘thermoregulatory insufficiency hypothesis’ postulates that animals (especially medium- and small-sized birds and mammals) struggle to maintain body temperature during cold nights [26,27]. This hypothesis predicts that group-living animals (i) would require more time in their sleeping huddle after cold nights, and (ii) larger groups would start their day earlier than smaller groups, as larger sleeping huddles may conserve body temperature better.

Here, we used a medium-sized desert specialist bird (Arabian babbler *Argya squamiceps*) as a model to study the determinants of daily activity onset and termination and the limitations of these temporal adjustments, also in relation to the long-term warming of the species’ habitat.

Arabian babblers (adult body mass: 66–79 g) are diurnal songbirds inhabiting the hyper-arid hot deserts of the Arabian Peninsula, Sinai Peninsula, Palestine and Israel [28]. These omnivorous birds primarily feed on a diversity of arthropods [29,30]. They live in cooperatively breeding stable groups of 2–20 members that occupy stable territories year-round, each including a handful of regular roosting trees [31]. In the evenings, group members usually engage in foraging until arriving at the roosting tree, where they settle into a sleeping huddle along an inner branch [31,32]. In the mornings, group members resume foraging soon after leaving the roosting tree. Only seldom do the birds first perform non-foraging behaviours in the mornings, such as a group ‘dance’ [33]. Under laboratory and resting conditions, Arabian babblers exhibited enhanced thermal conductance and a broad, elevated thermal neutral zone (31.6–40°C) [34]. However, this likely overestimates their thermoregulatory threshold during active foraging on hot ground and under direct solar radiation [35,36]. Indeed, even desert specialists like Arabian babblers struggle during the extreme arid and hot summers in the Arava desert: they halt breeding [37], fail to maintain body mass [25,38] and routinely rest under shaded areas around mid-day [39].

The specific goals of the current study were threefold. First, we utilized a long-term climatic dataset (1994–2025) to reconstruct the daily and annual temperature patterns at our study site (the Shezaf Nature Reserve, Arava Desert, Israel), thereby identifying daily and yearly periods of relatively cooler or warmer conditions.

Second, we investigated the effect of different climatic, ecological and social factors on the onset and termination times of daily activity. Specifically, we tested the effect of the following. (i) Ambient temperature: see predictions for the ‘heat’ and ‘thermoregulatory insufficiency’ hypotheses above. (ii) Rain: if rain disturbs sleep quality by wetting and thus cooling the birds [40,41] and/or by creating noise and vibration on the canopy [42,43], we predicted that the birds would wake up later following rainy nights. (iii) Wind: if wind impairs sleep quality through noise and vibration [42,43], we predicted that the birds would wake up later after windy nights. (iv) Night/day duration: if activity timing is adjusted to meet sleeping needs, we predicted that the birds would begin their daily activity later after short nights and terminate it earlier following long days. (v) Number of birds per roost (see above predictions) and (vi) group split: if social cohesion is important, we predicted that when the group split to roost on two trees, the separated parties would leave their roosting trees earlier to re-unite soon in the morning (compared to most nights when the entire group roost on one tree). Similarly, we predicted that a group that splits will terminate its daily activity later in the evening due to within-group negotiation about where to roost.

Third, we quantified longitudinal temperature change in our study site (1994–2025) while focusing on summers as the harshest annual period. We subsequently compared times of activity onset between the first (2012 and 2014) and last study summers (2024–2025) to examine whether climatic warming correlates with earlier activity onset.

## 2. Methods

### (a) Study population

We studied a wild population of Arabian babblers in the Shezaf Nature Reserve and its surrounding areas in the Arava Desert in Israel (30.73° N, 35.27° E). Birds were individually marked with a unique combination of three coloured plastic rings and a numbered metal ring. The total weight of rings is 0.34 g, which is ~0.5% of the mean body mass of adult females [38]. Habituation of the birds to human researchers enables observing their roosting behaviour from a distance of 4–10 m without eliciting alarm calls [44].

### (b) Behavioural observations

We conducted behavioural observations from November 2011 to July 2012 (*n* = 123, of which 16 during summers), from February 2014 to June 2014 (*n* = 29, of which 4 during the summer) and from August 2024 to July 2025 (*n* = 144, of which 40 during summers). In the mornings, we arrived at the roosting tree at dark and recorded when the first bird left the group’s roosting huddle to do a new activity (e.g. foraging, sentinel behaviour). In the evenings, we followed the focal group starting from 1 to 2 h before dusk until it reached its roosting tree. To ensure the birds did not change the roosting tree later, we recorded the time 2 min after the last bird arranged in the roosting huddle. We also recorded whether the group roosted in one or two different trees. Group IDs and times were recorded on a smartphone to the nearest second using CyberTracker software (www.cybertracker.org). Arabian babbler groups often include a dominant breeding pair [45], and when a member of this dominant pair changed, the group was given a new ID.

### (c) Data preparation

Temperature, rain and wind speed data were imported from the Israel Meteorological Service (https://ims.gov.il/en/data_gov), which operates a meteorological station at our research site (‘Hazeva’ station). Mean air temperature (0.1°C accuracy) and gust wind speed (m s^−1^, maximal wind speed during 2 s) were recorded every 10 min between 31 January 1994 and 27 July 2025 (*n* = 11 319 days). Total rainfall (0.1 mm accuracy) was recorded every 10 min between 1 January 2009 and 27 July 2025. Daytime was defined as the period between morning and evening civil twilights of the focal date (i.e. when the centre of the sun is geometrically 6° below the horizon). Night-time was defined as the period starting at the evening civil twilight of the previous day and ending at the morning civil twilight of the focal date.

A day was considered to last from one evening civil twilight to the next. Minimum and maximum daily temperatures were identified by calculating a rolling average for each three consecutive temperature records (i.e. a total of 30 min) and identifying the minimum and maximum values among these 30 min averages. The time between morning civil twilight and minimum daily temperature and the time between evening civil twilight and maximum daily temperature were calculated after all dates with >1 missing temperature record were removed (*n* = 11 256 days).

### (d) Statistical analyses

Data preparation and statistical analyses were conducted in R (v. 4.2.2 [46]). Models’ assumptions were examined using the package DHARMa (v. 0.4.7 [47]). Collinearity between fixed effects was examined by their variance inflation factor [48]. *R*^2^ was calculated using the R package part *R*^2^ (v. 0.9.2 [49]). Comparison between mixed effect models was made using the drop1 function with the test argument ‘F’ for linear models and the test argument ‘*Chi2*’ for mixed effect models [50]. ‘BOBYQA’ nonlinear optimization was used to aid convergence of models [51].

*Behavioural analyses*. To allow comparison throughout the year, times of morning/evening civil twilight were used as reference points for when the group started/ended its daily activity, respectively. The package ‘suncalc’ (v. 0.5.1 [52]) was used to extract the times of morning civil twilight (keep: ‘dawn’) and evening civil twilight (keep: ‘dusk’; lat: 30.73 and lon: 35.27).

To explain variation in times of daily activity onset, we used linear mixed models (lme4 package v. 1.1-35.1 [53]). The response variable was the number of minutes between when the first bird left the group’s roosting huddle and morning civil twilight. The fixed effects were: (i) rain during night-time (yes ≥ 0.1 mm/no = 0 mm); (ii) mean gust wind speed during the night-time; (iii) group split to roost in two trees (yes/no); (iv) number of birds roosting in each tree; (v) night-time duration (h); and (vi) two aspects of recently experienced air temperature: (a) mean and (b) maximum temperature during the last 24 h [7]. Air temperature was used since thermal neutral zone is often quantified with air temperature [35], and in our study site, it is correlated with grass temperature (i.e. temperature at 5–10 cm above ground). Since we predicted a nonlinear effect when temperature considerably exceeds the animal’s thermoneutral zone, temperature predictors were modelled using a natural spline with one or two knots (‘splines’ package v. 4.2.2 [46]). Because both temperature terms correlated with each other and also with night duration, only one of these three terms was included in each model at a time (see model selection procedure below [54]).

To explicitly test the thermoregulatory insufficiency hypothesis, we repeated this morning model using mean night temperature (i.e. from dusk to dawn) instead of the last 24 h mean temperature, and restricted the analysis to relatively cold nights below 20°C (*n* = 136 mornings and 23 groups).

To explain variation in times of termination of daily activity, we used linear mixed models with the response variable being the number of minutes between the evening civil twilight and when all birds were clumped in a roosting huddle for 2 min. The fixed effects were: (i) rain during daytime (yes ≥ 0.1 mm/no = 0 mm); (ii) group split to roost on two trees (yes/no); (iii) number of birds roosting in each tree; (iv) day-time duration (h); and (v) mean or maximum temperature during the last 24 h.

All three behavioural models included the Group ID as a random effect. The sample size of the morning model was 250 mornings and 27 groups (2 mornings without climatic data were excluded from the model). The sample size of the evening model was 151 evenings and 21 groups (1 evening without data on group split was excluded from the model). Repeating the morning and evening models using only the data from the first study period (2011–2014) or only data from the second study period (2024–2025) yielded similar results to the models that included all years (electronic supplementary material).

**Figure 2.**
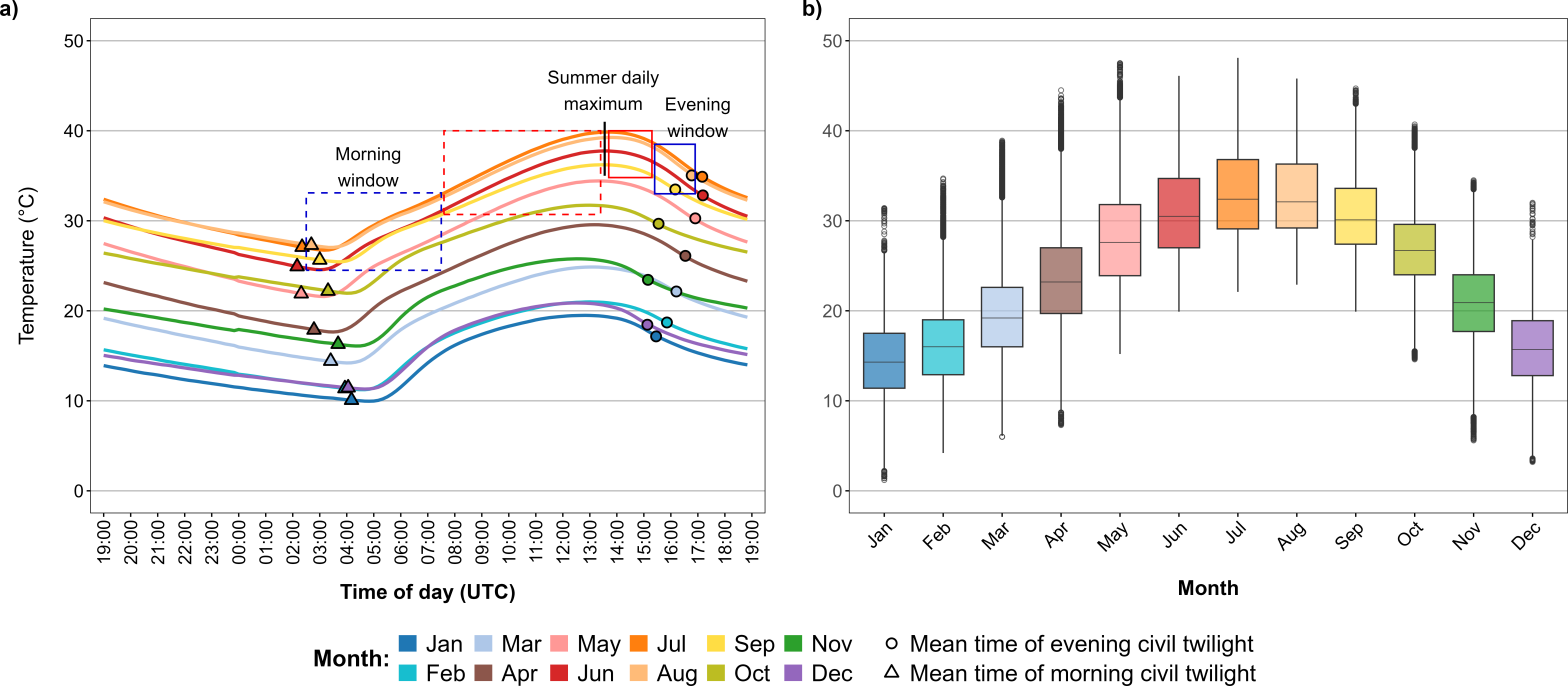
(a) Daily temperature structure at the Shezaf Nature Reserve (Israel). Mean air temperature across a 24 h cycle by month. The *x*-axis presents UTC (Coordinated Universal Time). The vertical black line represents the mean time of maximum daily temperature during the summer period (June–September). Each dashed square bounds the temperature during one half of the time between the morning civil twilight and the time of daily maximum temperature (5.5 h each). Each solid square bounds the temperature during one half of the time between the time of maximum daily temperature and evening civil twilight (1.5 h each). (b) Yearly temperature structure at the Shezaf Nature Reserve. Boxes represent the interquartile range (25th–75th percentile), horizontal bars indicate the median and whiskers extend to the minimum and maximum values, excluding outliers. Individual points beyond the whiskers represent outliers (*n* = 1 593 330, 10 min interval temperature records between 1994 and 2025).

*Climatic analyses*. We constructed three complementary linear models to test different aspects of temperature change during 1994–2024. The models differ in their response variables, which were: (i) mean daily air temperature (in the ‘all year’ model); (ii) mean temperature during the daily morning time window in the summer months (June–September; hereafter the ‘summer mornings’ model); and (iii) mean temperature during the daily evening time window in the summer months (hereafter the ‘summer evening’ model). The morning and evening time windows were defined to represent the cooler period in each half of the daytime. To this end, the daytime was divided before and after the time of maximum daily temperature (figure 2a). Next, the period between morning civil twilight and the time of maximum temperature was divided into two halves (each of 5.5 h) and the cooler half was defined as the ‘morning window’ (i.e. the earlier half). Similarly, the period between the time of maximum daily temperature and evening civil twilight was divided into two halves (each of 1.5 h), and the cooler half was defined as the ‘evening window’ (i.e. the latter half; figure 2a). Note that these time windows are also when Arabian babblers are most active during the summer [39]. Using a morning window of 3 or 4 h and an evening window of 2 h resulted in similar results. All three climatic models had the same two fixed effect predictors: (i) year and (ii) date index as a continuous variable varying between 0 (1 January) and 1 (31 December). Since we predicted a nonlinear effect of both fixed effects (also due to the large sample size that allows detecting relatively small nonlinear changes), we modelled each term using a natural spline with 1–10 knots and chose the model with the lowest BIC. The sample size of the all-year model was 10 857 days and 30 years (1995–2024). The sample size of the morning model was 3756 mornings and 31 years (1994–2024). The sample size of the evening model was 3750 evenings and 31 years (1994–2024).

### (e) Model selection

The morning and evening models have gone through a double-stage model selection process. The first stage identified the model that explains maximum variation in the most parsimonious way. To this end, we ran models with all possible combinations of fixed effects (without interactions) and identified all models with BIC values ≤2 from the model with the lowest BIC value. Only the set of morning models included more than one model (three models with similar compositions of fixed effect), and we thus chose the model with the lowest AIC out of this subset of lowest BIC models. The second stage tested the statistical significance of each term in the best model by comparing its full version with its reduced version that misses a different fixed effect at a time [50].

## 3. Results

### (a) Daily and yearly temperature structures

The Shezaf Nature Reserve has a unimodal temperature structure at the daily and yearly levels (figure 2a). Daily minimum temperature (mean ± s.e.: 18.8 ± 0.06°C) occurs 42.3 ± 1.6 min (mean ± s.e.) after morning civil twilight (*n* = 11 072 days). Afterwards, the temperature increases continuously until reaching its daily maximum (mean ± s.e.: 30.6 ± 0.07°C) 183.9 ± 0.7 min before evening civil twilight (*n* = 11 072 days). At the yearly level, air temperature reaches its highest value during July and August. We considered the period from June to September as summer because the median/maximum daily temperature often exceeds 30°C (figure 2b)/40°C (figures 1 and 2b), respectively.

### (b) Onset and termination of daily activity

**Figure 3.**
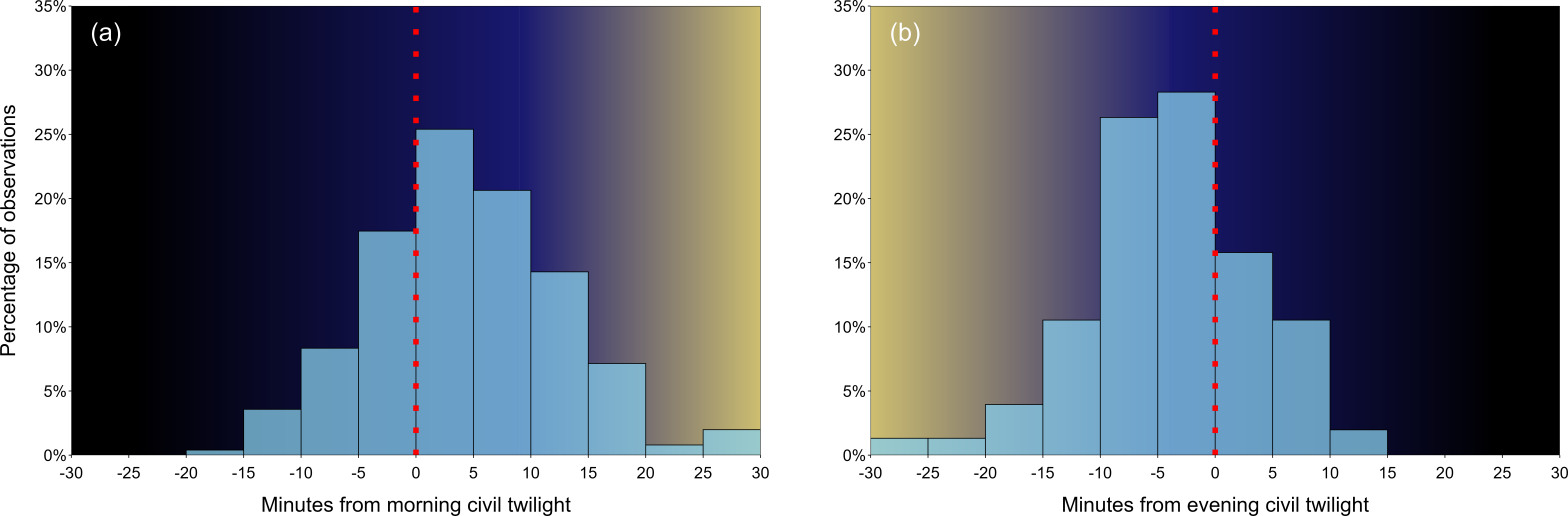
Times when Arabian babbler groups (a) left their roosting huddle in the mornings (*n* = 252 mornings) and (b) gathered in a roosting huddle in the evenings (*n* = 152 evenings) in relation to civil twilights. The dotted red lines at *x* = 0 represent the time of civil twilight. The gradient-coloured backgrounds are proximate illustrations of sunlight availability during different time phases.

**Figure 4.**
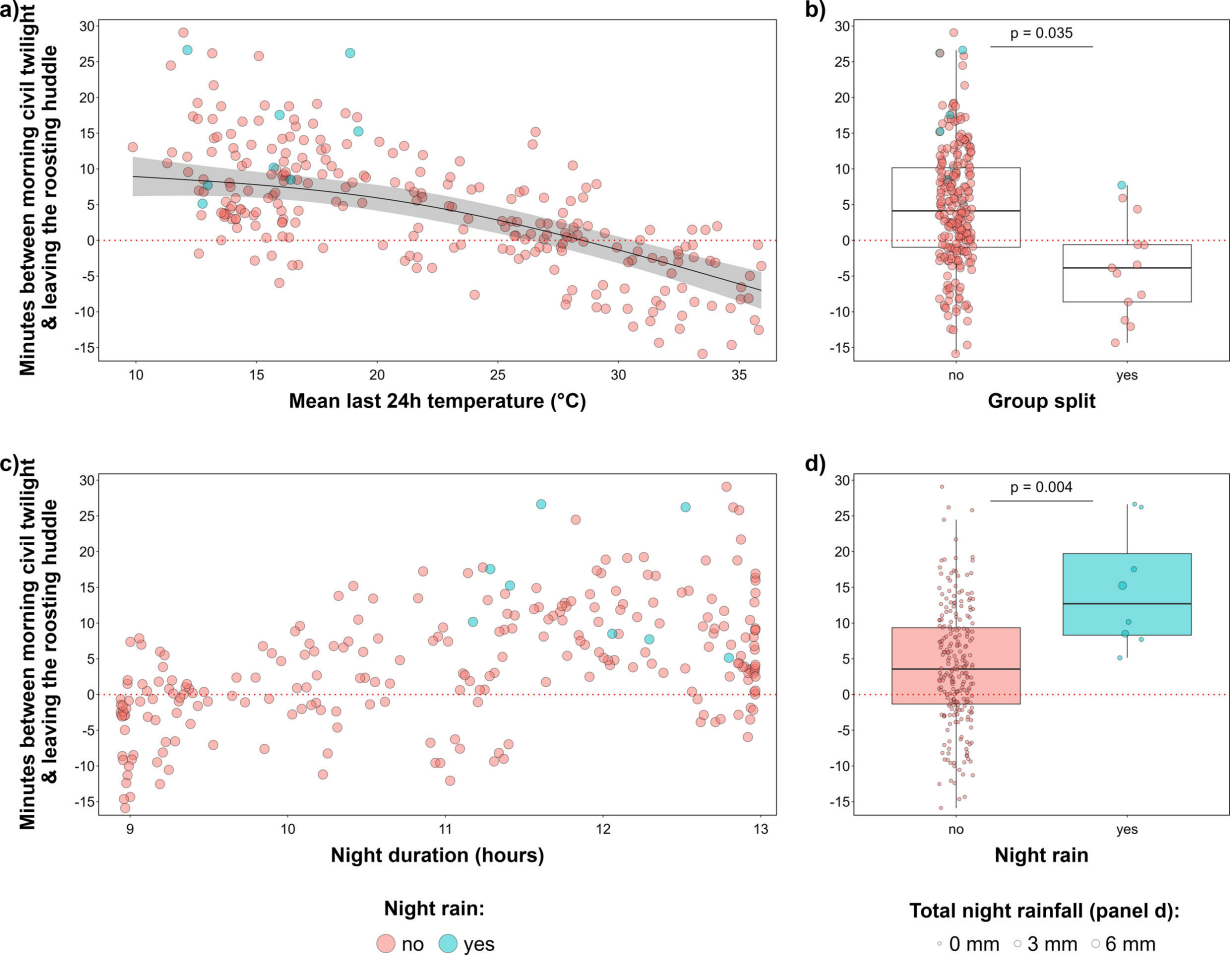
(a) The effect of the last 24 h mean temperature, (b) group roosting in one or two trees, (c) night duration and (d) rain during the night on time leaving the roosting huddle (*n* = 250 mornings). The dotted red lines (at *y* = 0) denote the time of morning civil twilight. In panel (a), the black lines and grey shaded areas represent the linear mixed model’s predicted regression and 95% confidence intervals. In panels (b) and (d), the horizontal bars represent the median, boxes represent the 25% and 75% quartiles and the vertical lines indicate the minimum and maximum values (points outside the lines are outliers). Predicted values are not drawn for night duration, as it was not included in the best model.

Arabian babbler groups started and terminated their daily activity within a 45 min time window around the morning and evening civil twilight (figure 3). In the mornings, groups left their roosting huddle earlier as the mean temperature during the last 24 h increased (figure 4a), and when the group split to roost in different trees (figure 4b). In contrast, rain during the night was associated with delayed departure from the roosting huddle (figure 4d, table 1a). Night duration (figure 4c), gust wind speed and the number of roosting birds were not included in the best model. In the model including only cold nights (i.e. mean night temperature ≤20°C), mean night temperature and group size had no effect on activity onset (delta BIC: 6 from the best model).

**Table 1.** Results of linear mixed models.

**(a) best morning model**					
**response variable**: time (min) between morning civil twilight and when the group left its sleeping huddle					
**random effect**: group ID, *n* = 250 mornings, 27 groups					
term	estimate	s.e.	*χ* ^2^	d.f.	*p*
intercept	8.95	1.41			
mean last 24 h temperature:			119.74	2	<0.001
knot 1	−12.60	2.72			
knot 2	−14.91	1.25			
night rain (yes)	5.06	1.76	8.30	1	0.004
group split (yes)	−2.98	1.42	4.45	1	0.035

(b) best evening model					
**response variable**: time (min) between evening civil twilight and when the group gathered in a sleeping huddle					
**random effect**: group ID, *n* = 151 evenings, 21 groups					
term	estimate	s.e.	*χ* ^2^	d.f.	*p*-value
intercept	−20.69	2.30			
mean last 24 h temperature	0.44	0.07	38.74	1	<0.001
group split (yes)	8.83	2.13	16.68	1	<0.001
birds in the roosting site	0.71	0.26	7.69	1	0.006

**Figure 5.**
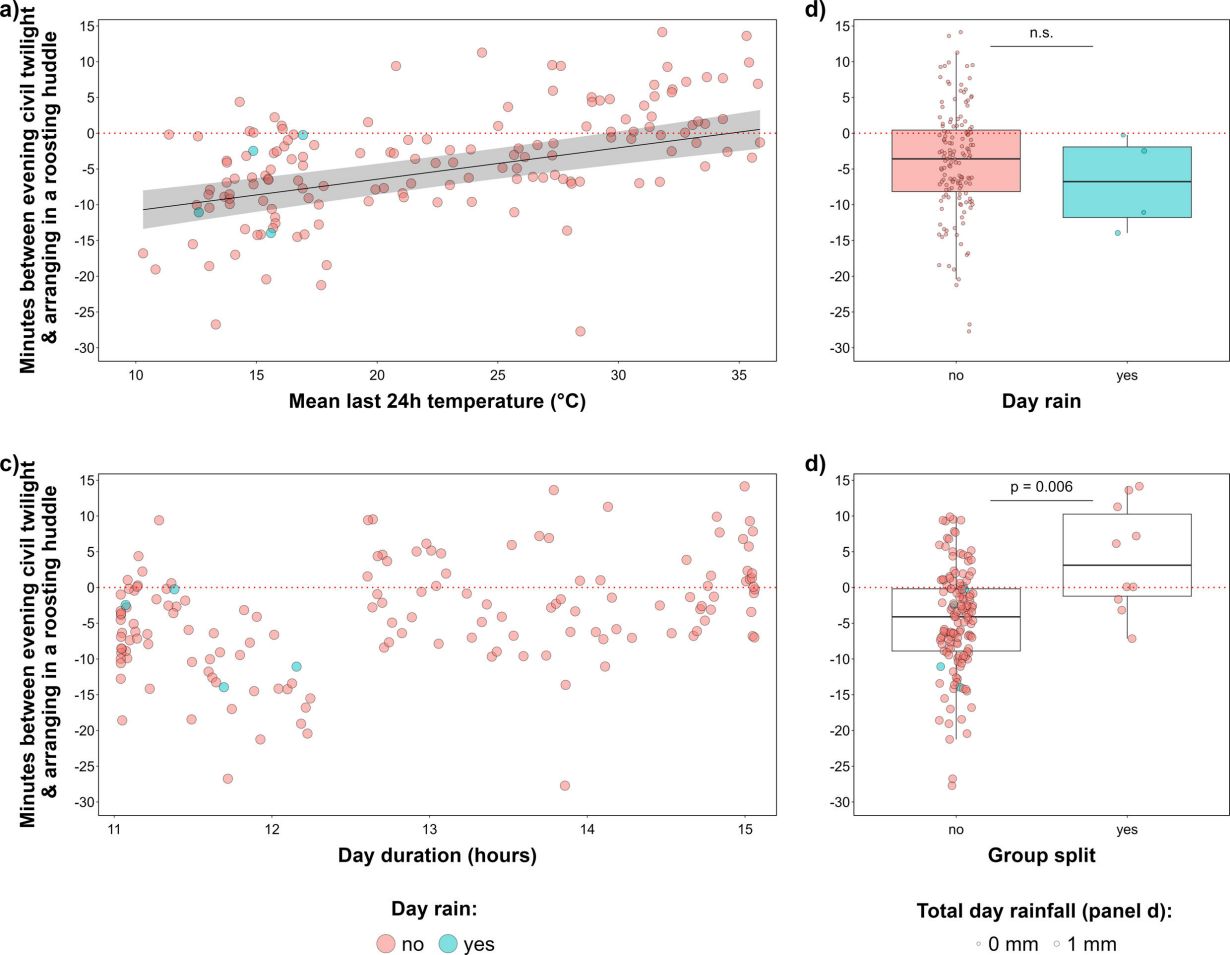
(a) The effect of the last 24 h mean temperature, (b) group roosting in one or two trees, (c) day duration and (d) rain during the day on time Arabian babbler groups gathered in the roosting huddle for sleep (*n* = 151 evenings). The dotted red lines (at *y* = 0) denote the time of evening civil twilight. In panel (a), the black lines and grey shaded areas represent the linear mixed model’s predicted regression and 95% confidence intervals. In panels (b) and (d), the horizontal bars represent the median, boxes represent the 25% and 75% quartiles and the vertical lines indicate the minimum and maximum values (points outside the lines are outliers). Predicted values are not drawn for the day duration because it was not included in the best model.

In the evenings, Arabian babbler groups gathered in their roosting huddle later as the mean temperature during the last 24 h increased (figure 5a), when the group split to roost in two trees (figure 5b), and slightly later with increasing group size (table 1b). Day duration (figure 5c), gust wind speed, and rain during the day (figure 5d) were not included in the best model.

### (c) Climate change and activity onset

During the last three decades, temperatures at the Shezaf Nature Reserve have increased throughout the year (*F*-statistic = 63.99, d.f. = 8, *p* < 0.001). Specifically, during summers, there was a significant temperature increase even during the cooler morning (*F*-statistic = 72.26, d.f. = 8, *p* < 0.001; electronic supplementary material, figure S1a) and evening time windows (*F*-statistic = 62.15, d.f. = 3, *p* < 0.001; electronic supplementary material, figure S1b).

During the first summers of the study (2012 and 2014), Arabian babblers in the reserve initiated daily activity 0.3 ± 0.8 min *after* morning civil twilight (mean ± s.e., *n* = 20 mornings). However, during the last summers of the study (2024 and 2025), the mean time of activity onset was 8.5 ± 0.7 min *before* morning civil twilight (*n* = 30 mornings; figure 1b).

## 4. Discussion

We studied a desert specialist bird species as a model for the interplay between behavioural responses to ambient heat and ongoing climate change. Our key behavioural findings are that (i) Arabian babblers started and terminated their daily activity within ~45 min time windows around the morning and evening civil twilights, but were never active during night-time (figure 3), and (ii) the birds initiated daily activity earlier and terminated it later in response to recently experienced heat (figures 4 and 5). These behavioural responses to ambient heat are discussed in relation to our finding that (iii) summers in the Arava desert have become hotter in the past decades, and (iv) at the same time, Arabian babblers advanced the onset of daily activity by ~8.8 min before the morning civil twilight.

### (a) Onset and termination of daily activity

We demonstrate a strict avoidance of night-time activity by Arabian babblers (figure 3). Activity onset around morning civil twilight seems common among songbirds [17,18]. Before the morning civil twilight and after the evening civil twilight, less indirect sunlight is available. Avoidance of activity in the dark may therefore be driven by two non-mutually exclusive factors. First, predation risk is higher since most desert predators are nocturnal (e.g. owls [55]*,* snakes [56], carnivores [14]). Second, foraging may be inefficient due to sensory limitations in the dark and/or scarcity of invertebrate prey under the colder night temperatures [57]. The poor light conditions before/after the morning/evening civil twilights thus seem an impassable barrier for earlier/later activity of diurnal species, respectively.

### (b) Adjustment of activity timing in response to recently experienced heat

Air temperature during the last 24 h had a considerable explanatory power of the variance in the onset (part *R*² = 0.37) and termination (part *R*² = 0.13) times of daily activity. The thermoregulatory insufficiency hypothesis was not supported by our data. First, night temperature had no considerable effect on daily activity onset when considering relatively cold nights (≤20°C). Second, although larger groups may maintain warmer roosting huddles, they did not start their daily activity earlier than smaller groups. These negative results align with previous research showing that Arabian babblers do not perform more morning ‘huddle dances’ in colder seasons [33,58], have never been observed basking by the authors, and are consistent with similar negative evidence reported in other desert songbirds [26].

In contrast, Arabian babblers responded to recently experienced heat by advancing their daily activity onset (figure 4a) and delaying their evening activity termination (figure 5a). In birds, extreme temperatures negatively affect inhibitory control [5,6], reproduction [59,60], metabolic rates and food intake [61,62], while increasing oxidative damage [63] and dehydration [64]. Specifically in Arabian babblers, summer heat leads to a reproductive pause [37], and a failure to compensate for the nightly mass loss leads to unsustainable body mass [25,38]. We propose that temporal adjustments of daily activity in response to recently experienced heat are beneficial in two additive ways. At the proximate level, the birds may overcome heat-induced energy deficiency by resuming foraging earlier in the mornings and continuing foraging as late as possible before the night fast. Concurrently, these temporal adjustments are also beneficial by extending foraging time under cooler temperatures because the coolest day periods in deserts are typically after/before the morning/evening civil twilight, respectively (figures 1 and 2).

While temporal adjustments of ~45 min may seem minor, they can be important for fully utilizing the daily periods with cooler temperatures during harsh desert summers. For example, assume a 5.5 h foraging session in the summer of 2024, starting either 10 min before or after the morning civil twilight (i.e. the 330 min session will end 320 or 340 min after civil twilight, respectively). In the first scenario, the animal will forage for the first 20 min under an average air temperature of 27.7°C, while in the second scenario, it will forage for the last 20 min under 34.7°C (in both scenarios, the other 310 min will occur during the same hours and temperatures). Given that the foraging efficiency of other desert songbirds considerably declines above ~32–35°C [7,61], adjustment of activity timing can facilitate more efficient foraging with less energy expenditure on thermoregulation. We thus suggest that, while the proximate reason for temporal activity adjustment in Arabian babblers is a response to heat-induced hunger, its primary advantage may lie in extending foraging time under cooler temperatures. This may join other behaviours the species may employ to mitigate heat stress and were not covered in the current study, as for instance, changes in foraging strategies to more shaded areas [16,61]. ince desert endotherms are under selective pressures to utilize the cooler parts of the day [8,10–12,65], it is reasonable to predict that similar temporal adjustments occur among other diurnal desert songbirds.

### (c) Temporal adjustments in response to other social and ecological factors

The splitting of the social group to roost in different trees was associated with later termination and earlier onset of daily activity (figures 4b and 5b). Group splitting in the evenings typically involves repeated movements between potential roosts, accompanied by contact calls, suggesting difficulty in reaching a collective decision regarding the roosting site [66]. Early in the next morning, the separated parties exchange contact calls and engage in sentinel behaviour to gather visual information to coordinate reunion [67]. These time-consuming behaviours probably reflect the importance of spatial cohesion among Arabian babbler group members that almost always stay within view of each other [68].

Rain during the day was not associated with adjustment of activity termination (figure 5d), but rain during the night was associated with delayed activity onset (figure 4d). These findings support the hypothesis that rain prolongs sleep needs because it impairs sleep quality, probably by wetting and cooling the animals [40,41].

In contrast to the hypothesis that times of activity onset and termination are influenced by sleep requirements, Arabian babblers initiated daily activity earlier after the shorter summer nights (figure 4c) and terminated it later after the longer summer days (figure 5c).

### (d) Climate change and limitations of activity time adjustments

For non-migratory desert species, the summer period often presents the harshest annual period for reproduction and survival [69–71]. Summers in the Shezaf Nature Reserve have nevertheless become considerably hotter over the past decades (figure 1; electronic supplmentary material, figure S1). This deterioration of climatic conditions may partly explain the decline in our Arabian babbler population during the last 40 years [72,73] and may be similar in other Middle Eastern desert songbird populations [20,74].

The summer activity of diurnal desert birds is naturally confined to the relatively cooler early morning and late evening periods [8,39]. We show, however, that climate change is continually narrowing these time windows. It thereby drives diurnal desert endotherms into a ‘heat versus dark trap’ where, on the one hand, they experience heat constraints if active more into the mid-day, but on the other hand, may suffer elevated predation and inefficient foraging if active more into the night-time (figure 1) [14,75]. The severity of this heat versus dark trap would depend on how much individuals of a certain species can advance their activity onset from its current time (e.g. civil twilight versus sunrise) before it becomes too dark. For example, diurnal desert mammals that start daily activity after sunrise are predicted to have more temporal leeway to advance activity before reaching night-time (e.g. Brants’s whistling rat *Parotomys brantsii* [65]*,* dwarf mongoose [12]). In contrast, songbirds who already start their daily activity around morning civil twilight [8,17,18] may have less leeway. Our Arabian babbler population exemplifies this constraint with an advancement of ~8.8 min in the onset of summer daily activity during the past decade, during which the habitat continued to warm (figure 1). Given the poor light conditions before morning civil twilight, it remains unclear how much further the birds can advance their daily activity in response to the continued warming of the Arava Desert before facing excessively high nocturnal predation risk and inefficient foraging. Taken together, our behavioural and climatic results suggest that other diurnal desert songbirds may likewise have already utilized a considerable extent of their behavioural plasticity with regard to the onset and termination of daily activity.

## Data Availability

The datasets used for this study and the results of all analyses are provided in the electronic supplementary material [76].
